# Dr. Louis E. Krombien

**Published:** 1903-01

**Authors:** 


Dr. Louis E. Krombien, of Buffalo, (U. of B., '60), died at his home in this city December 7, 1902, aged 76 years. His death was sudden and was believed to be due to sequelæ of an attack of grip, from which he had recently suffered, and that his age and feebleness proved unequal to the strain.

Dr. Krombien had been in practice here from his graduation and had many attached friends. Of late years his work had been somewhat limited by his health, which was not robust, but he did not retire from practice. He was an amiable, modest man and mingled but little with the world, giving his entire attention to his own affairs.

